# Uncovering Driver DNA Methylation Events in Nonsmoking Early Stage Lung Adenocarcinoma

**DOI:** 10.1155/2016/2090286

**Published:** 2016-08-17

**Authors:** Xindong Zhang, Lin Gao, Zhi-Ping Liu, Songwei Jia, Luonan Chen

**Affiliations:** ^1^School of Computer Science and Technology, Xidian University, Xi'an 710000, China; ^2^Department of Biomedical Engineering, School of Control Science and Engineering, Shandong University, Shandong 250061, China; ^3^Key Laboratory of Systems Biology, Institute of Biochemistry and Cell Biology, Shanghai Institutes for Biological Sciences, Chinese Academy of Sciences, Shanghai 200031, China; ^4^Institute of Industrial Science, University of Tokyo, Tokyo 153-8505, Japan; ^5^School of Life Science and Technology, ShanghaiTech University, Shanghai 201210, China

## Abstract

As smoking rates decrease, proportionally more cases with lung adenocarcinoma occur in never-smokers, while aberrant DNA methylation has been suggested to contribute to the tumorigenesis of lung adenocarcinoma. It is extremely difficult to distinguish which genes play key roles in tumorigenic processes via DNA methylation-mediated gene silencing from a large number of differentially methylated genes. By integrating gene expression and DNA methylation data, a pipeline combined with the differential network analysis is designed to uncover driver methylation genes and responsive modules, which demonstrate distinctive expressions and network topology in tumors with aberrant DNA methylation. Totally, 135 genes are recognized as candidate driver genes in early stage lung adenocarcinoma and top ranked 30 genes are recognized as driver methylation genes. Functional annotation and the differential network analysis indicate the roles of identified driver genes in tumorigenesis, while literature study reveals significant correlations of the top 30 genes with early stage lung adenocarcinoma in never-smokers. The analysis pipeline can also be employed in identification of driver epigenetic events for other cancers characterized by matched gene expression data and DNA methylation data.

## 1. Introduction

As a leading cause of death worldwide, lung cancer is mainly attributed to smoking in both men and women [1, 2], of which the most common histological subtype is adenocarcinoma. However, as smoking rates decrease, proportionally more cases occur in never-smokers [3]. Lung adenocarcinoma in never-smokers shows obvious distinctions in clinical and molecular mechanism to those cigarette smoking [4]. Both genetics and epigenetics in cancer genomes have been suggested to account for the development of lung adenocarcinoma.

As one of the vital epigenetic mechanisms, DNA methylation regulates gene expression without alterations in DNA sequence [5, 6] and plays key roles in X chromosome inactivation, genome stability, chromatin structure, embryonic development, differentiation, and maintenance of pluripotency in normal somatic cells [7, 8]. Genome-scale methylation-profiling techniques have confirmed the existence of widespread aberrations of DNA methylation patterns in human cancer genome [9–12]. Studies of DNA methylation have suggested that both global DNA hypomethylation and gene-specific hypermethylation may contribute to the initiation and progression of tumorigenesis, as well as gene body methylation [13–15]. It is challenging but of great significance to distinguish genes whose methylation changes are crucial in cancer occurrence, progression, or metastasis from genes whose methylation changes merely have effects on the process of tumorigenesis in cancer research and therapy [13]. Unlike somatic mutations in the genome, DNA methylation is inherently reversible and serves as potential drug targets in cancer intervention [16, 17].

Numerous studies have focused on discovering genes whose DNA methylation potentially plays key roles in tumorigenesis of lung adenocarcinoma, including integration of genome-scale DNA methylation and gene expression [18–21]. The main idea of these works is to search genes whose gene expression fluctuations are highly correlated to DNA methylation changes. However, there is a deficiency derived on the complexity of the gene expression regulation. Both genetic and epigenetic alterations can contribute to gene expression as well as other transcriptional factors in sophisticated manners in complex diseases [22, 23]. In tumors, a differential gene expression may be induced by an aberrant DNA methylation in the promoter of the gene but also may be a consequence regulated by its upstream genes in regulatory mechanisms. These appeal to a great attention in uncovering driver DNA methylations, which play major roles in methylation-associated gene silencing and drive malignant transformation [5, 13]. In this work, we refine the generalized description of driver methylation as two properties. (1) Driver DNA methylation should induce distinctive expressions in tumors with differential DNA methylation (T-DM) when compared to expressions in matched adjacent nontumor (normal) and tumors with nondifferential DNA methylation (T-NDM), and (2) driver methylation should induce a distinct regulation module in the network perspective. The first property guarantees the major role of DNA methylation in the regulation of gene expression, while the second property guarantees the functional effects of driver genes on tumorigenesis.

Focusing on genes differentially expressed among matched adjacent nontumors (normal), tumors with aberrant DNA methylation (T-DM), and tumors without aberrant DNA methylation (T-NDM), we integrate genome-wide DNA methylation data and gene expression data to uncover driver methylation events in never-smokers in early stage lung adenocarcinoma. Differential network analyses show significant changes of DNA methylation-responsive modules in network topology across normal, T-DM, and T-NDM, which imply potential mechanisms of identified driver genes underlying the tumorigenesis.

## 2. Materials and Methods

### 2.1. Data Sets

Both the DNA methylation data and gene expression data are downloaded from NCBI Gene Expression Omnibus (GEO) with accession number GSE32867 [18]. The series contains 59 samples with paired genome-scale DNA methylation profiling and gene expression. Stage I and stage II are merged as early stage and stages III-IV are labeled with late stage [18]. After removing noisy data [18], 22 samples are labeled with “never smoking” and “early stage” simultaneously. Paired DNA methylation data and gene expression data of these 22 samples are collected to further analysis. Probes in gene expression data are firstly mapped to Entrez gene ID and expression values sharing same Entrez gene IDs are averaged among samples.

### 2.2. Schematic Overview of the Analysis Pipeline

**Figure 1:**
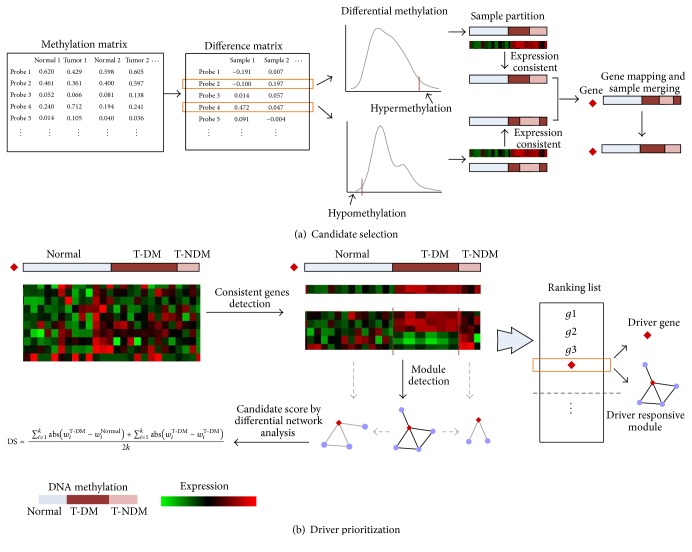
Schematic overview of the pipeline proposed in this work. (a) Candidate gene selection. Methylation matrix of continuous beta values is converted into difference matrix and discretized by kernel distribution function, which partition samples into normal, T-DM, and T-NDM. Probes are mapped to genes after noise filtering and genes passing the consistent test are collected as candidate driver genes. (b) For each candidate gene, a subset of DM responsive genes is collected and DM responsive modules are constructed by the CLR method. Candidate driver genes are ranked by differential scores derived on the differential network analysis.

The schematic overview of the analysis pipeline is shown in Figure 1, and detailed procedures are described in the following sections.

#### 2.2.1. Candidate Driver Gene Selection

Figure 1(a) shows a brief schematic overview of this procedure. The difference matrix is firstly created to measure differences of beta values of DNA methylation between tumor and normal. The kernel probability distribution with normal smoothing function is used to estimate the probability density distribution for each probe in the difference matrix (Figure 1(a)). The hypothesis is that the differences of beta values for given probes come from distributions with the mean 0 and unknown variances. The cumulative density function (CDF) is used to estimate the probability of a beta value falling within given interval. Hypermethylation and hypomethylation are determined by the upper bound CDF > 0.95 and the lower bound CDF < 0.05, respectively. For each probe, tumors are partitioned into two groups, tumors with differential methylation group (T-DM) and tumors without differential methylation group (T-NDM).

Then, the two-sample *t*-test is used to evaluate differential expression under conditions [24], and *p* values are adjusted by the procedure introduced by Storey [25]. The mapping from DNA methylation to gene expression is performed by shared Entrez gene ID. Probes remain if the mapped genes are differentially expressed in T-DM when compared to normal and T-NDM (adjusted *p* value < 0.05), which implies that the differential methylation of given probes in T-DM is more likely to induce significant expression changes. Probes mapping to same genes are removed if hypermethylation and hypomethylation coexist in more than 5 samples. Then samples in T-DMs and T-NDMs merge, respectively, by shared Entrez gene ID and serve as T-DM and T-NDM of the gene.

We then search for genes whose expressions are highly discriminative and consistent in T-DM when compared to normal and T-NDM. Many types of statistics, such as Wilcoxon score, Pearson correlation coefficient (PCC), or mutual information (MI), could be used to score the relationship between gene expression and class labels, and a *T*-score method is used in this work [26]. For a given gene, let *a* be the gene expression levels across samples with class *c* and the discriminative score *s*(*a*, *c*) is defined as the* t*-test statistic. To determine whether the discriminative level of the gene among groups is consistent, we permute the class *c* by 1000 times and obtain a background distribution of the discriminative scores *S*′(*a*, *c*) derived on the gene expression levels *a* and permuted class *c*′. Genes with significant values (*p* value < 0.05) among groups (normal versus T-DM and T-DM versus T-NDM) are considered differentially methylated and served as candidates for further analysis.

#### 2.2.2. Detection of DNA Methylation-Responsive Module

To construct the DNA methylation-responsive module for a candidate gene *g*, we firstly recognize a set of genes whose expressions are highly discriminative among groups defined by DNA methylation profiles of *g*. These genes are potentially responsive to aberrant DNA methylation of *g*.

The Context Likelihood of Relatedness (CLR) method [27] is used to assess regulatory relationships among these genes. CLR estimates MI for each pair of variables and corrects the MI via a background-corrected procedure. In particular, for mutual information *I*(*X*_*i*_; *X*_*j*_), CLR scores the relatedness between a pair of variables *X*_*i*_ and *X*_*j*_ by the joint likelihood measurement:

(1) $$\begin{array}{c} z_{ij}=\sqrt{z_{i}^{2}+z_{j}^{2}}, \end{array}$$

where

(2) $$\begin{array}{c} z_{i}=max\left(\;0,\frac{I\left(X_{i};X_{j}\right)-\mu_{i}}{\sigma_{i}}\;\right), \end{array}$$

where *μ*_*i*_ and  *σ*_*i*_ are the mean and standard deviation derived on the empirical distribution of MI between *X*_*i*_ and arbitrary variables *X*_*k*_  (*k* = 1,2,…, *n*) and  *I*(*X*_*i*_; *X*_*j*_) is the mutual information of *X*_*i*_ and *X*_*j*_.

CLR employs B-spline smoothing and discretization method [28] to estimate the MI for a pair of variables. However, it is time-consuming in this work under diversiform conditions and permutations. Thus, we use the following estimation method to calculate MI for pair of variables *X*_*i*_ and *X*_*j*_ [29]; that is,

(3) $$\begin{array}{c} I\left(\;X_{i};X_{j}\;\right)=-\frac{1}{2}\log\left(\;1-\rho^{2}\;\right), \end{array}$$

where *ρ* is the PCC of *X*_*i*_ and *X*_*j*_.

An experienced threshold *δ* is necessary when CLR is employed. A larger threshold results in a higher precision but a smaller size of responsive modules. The size of more than 70% modules is less than three when *δ* = 4.46, while the size of 80% modules is larger than 3 when *δ* = 4.46 and approximate ranking lists of top 30 genes are obtained when *δ* falls in the interval between 3.96 and 5.46. Thus, we set *δ* = 4.46 in this work.

#### 2.2.3. Scoring Candidate Driver Genes by Differential Network Analysis

Differential network analysis reveals dynamic changes of pathways and potential mechanisms in complex diseases including cancers [30]. For each candidate gene, we calculate CLR scores for edges in responsive modules under normal and T-NDM. Differential scores are calculated to estimate network differences among groups. The differential score (DS) is yielded by the following equation:

(4) DS=∑i=1kabswiT-DM−wiNormal+∑i=1kabswiT-DM−wiT-TDM2k,

where *w*_*i*_ is the CLR score of the *i*th edge and *k* is the number of edges in driver methylation-responsive module. Then candidate genes are prioritized by DS scores in descending order.

## 3. Results

We focus on the detection of differentially methylated genes which play key roles in tumorigenesis (“driver methylation gene”) and modules responsive to aberrant methylation of these genes. Rather than genes with consistent expressions to DNA methylation levels in whole tumors, we detect genes differentially expressed and consistent with DNA methylation in T-DM when compared to normal and T-NDM.

### 3.1. Identification of Candidate Driver Genes in Tumorigenesis

By integrating DNA methylation and corresponding gene expression data, the samples are partitioned into three groups (normal, T-DM, and T-NDM) for each gene (Figure 1(a)). Firstly, we remove genes that are not differentially expressed in T-DM when compared to normal and T-NDM. Then a permutation test is performed to determine the significance of the consistency of gene expression changes in T-DM when compared to T-NDM. To obtain a significant level of differences, we randomly permute T-DM and T-NDM and calculate differences. After 1000 times permutation, a background distribution of differences is constructed. After removing genes with the absolute mean beta value less than 0.1, 135 genes remain in the candidate list (see Supplementary File in Supplementary Material available online at http://dx.doi.org/10.1155/2016/2090286). We perform a functional enrichment analysis using DAVID [31, 32]. Of these 135 genes, 115 are annotated to GO terms including cancer-related functions such as response to stimulus, development process, cell differentiation, cell adhesion, cell growth and cell death, DNA repair, and apoptosis, which imply potential relationships between cancers and these 135 genes.

### 3.2. Detection Responsive Modules of Candidate Driver Genes

Biological network reveals cell's functional organization [33]. To characterize the functional implications of candidate driver genes in tumorigenesis, we detect modules responsive to differential methylation of candidate driver genes (Section 2). Totally, 130 of 135 modules have at least one edge when the threshold of CLR is set to 4.46, and the mean size of 130 modules is 15.

### 3.3. Prioritization of Candidate Driver Genes by Differential Network Analysis

We argue that a driver DNA methylation can induce not only a distinctive gene expression in T-DM, but also a distinctive module responsive to the alteration. We score each candidate driver gene by analysis of the differential level of the responsive module. Candidate driver genes are ranked by differential scores in descending order.

We testify the significance of the differential score to a background distribution derived from random permutations. For a given candidate driver gene, genes are randomly selected from its possible responsive genes with module size maintained, and a new module is constructed by CLR with *δ* = 4.46 as well as a differential score. A sequence of DS′ consisting of random differential scores is obtained after 1000 times random permutation. Of 135 candidate driver genes, 130 genes pass the test with *p* value < 0.01.

We also perform a differential network analysis of responsive modules under different CLR thresholds from 1.96 to 6.96 with step 0.5. Almost all modules obtain significant differential scores under CLR cutoffs (Supplementary File).  Table 1 lists details of top 30 genes.

## 4. Discussion

We build two lists as background to testify the accuracy of the ranked list. The first consists of genes that show absolute mean fold change larger than 0.2 in T-DM and literature annotated in lung cancer. Totally, 29 genes are contained in the first list and denoted as Standard_Lit. The other one comes from Selamat et al. of 76 genes [18]. In fact, this list is not very suitable because genes in Selamat et al. are confused with differentially methylated genes under smoking and late stage. Thus, we select genes covered by list from Selamat et al. and our list. Totally 19 genes are in the list and denote as Standard_Sel. Genes in these two lists are listed in Supplementary File.

**Figure 2:**
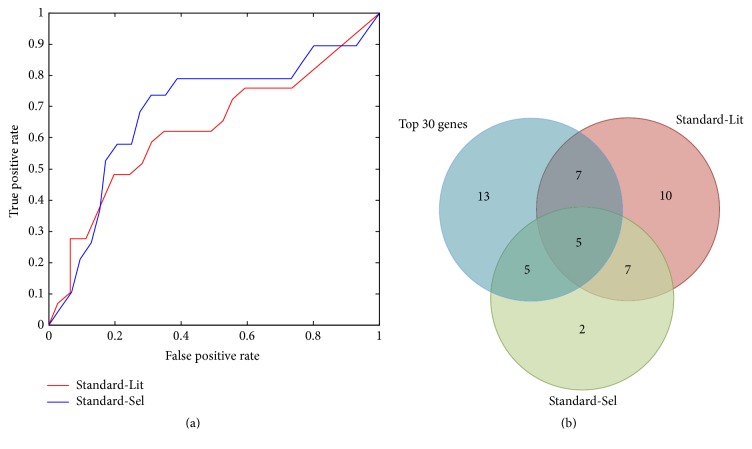
Comparison of the ranked list to two standard sets denoted by Standard-Lit and Standard-Sel. (a) ROC curves of our ranked list compared to Standard-Lit and Standard-Sel with AUC equal to 0.686 and 0.628, respectively. (b) Venn diagram showing the overlap of top 30 ranked genes in our list to Standard-Lit and Standard-Sel.

We test the accuracy of our list to Standard_Lit and Standard_Sel; Figure 2(a) shows the ROC curves with AUC = 0.686 and AUC = 0.628, respectively, which means that over half of genes in two standard lists are high-ranked in our list.  Figure 2(b) shows the overlaps of the top 30 genes in our list to Standard-Lit and Standard-Sel. For Standard-Lit, 12 of 29 genes are overlapped (Fisher exact test *p* value = 0.0018), while for Standard-Lit, 10 of 29 genes are overlapped (Fisher exact test *p* value = 2.67*E* − 04).

The ranked list is also validated by literature annotation. Of the top 30 genes, 27 genes are previously reported to be cancer-relevant, while 17 of them are lung cancer or non-small-cell lung cancer-related (Table 1).

We also annotate responsive modules of top 30 ranked genes to KEGG signaling pathways. Among them, responsive modules for 18 genes are enriched with KEGG signaling pathways with significance level *p* value < 0.01, which imply significant relations of these responsive modules to cancer processes (Table 2) and indicate potential mechanism changes induced by aberrant DNA methylation. The KEGG signaling pathways are collected from MsigDB [63, 64].

Of 30 top ranked genes,* FAM107A*,* MAMDC2*,* SOX17*,* TCF21*,* PTPRH*, and* CDO1* have been previously reported with aberrant DNA methylation in lung cancer [18, 34, 45, 46, 52, 57]. All these genes obtain higher occurrences (*n* > 19) in lung adenocarcinoma.* AGR2*,* CDH13*,* CRYAB*,* MX2*,* SH100P*, and* SH3GL2* are reported with aberrant gene expression [38, 40, 47, 54, 58, 59], while* AGR2*,* CDH13*, and* MX2* are of high occurrences in aberrant DNA methylation (*n* ≥ 18). Differential expression of these genes has been reported playing crucial roles in key pathways in tumorigenesis or serving as potential prognostic targets. With higher occurrences, the correlation of differential gene expression and aberrant DNA methylation of* AGR2*,* CDH13*, and* MX2* have been reported relevant to lung adenocarcinoma [18].

**Figure 3:**
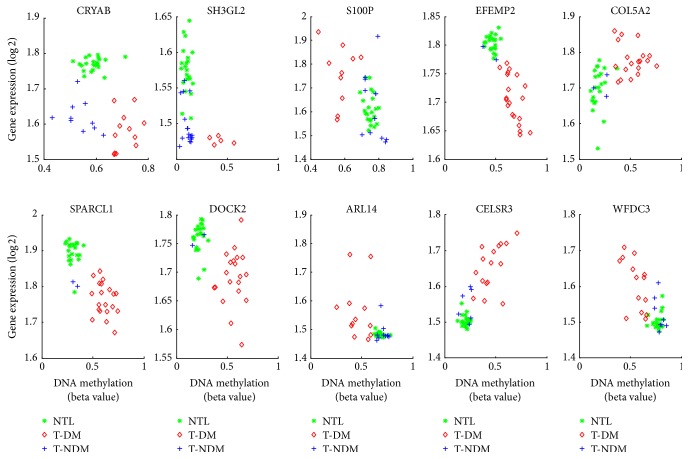
Genes show consistently significant changes in gene expression and DNA methylation in T-DM (red diamond) when compared to normal (green star) and T-NDM (blue plus). Results indicate different distributions of gene expression with altered DNA methylation in three groups of top ranked genes.

Alpha B-crystallin (*CRYAB*) is one of the important members of the small heat-shock protein family with aberrant DNA methylation occurring in 12 of 22 samples. The upregulated expression of* CRYAB* is reported relevant to the poor survival of patients with non-small-cell lung cancer (NSCLC) [38]. Interestingly, we find a contrary expression pattern in early stage lung adenocarcinoma in nonsmoking patients (Figure 3). A decreased expression is observed in both T-DM (*p* value = 8.20*E* − 11) and T-NDM (*p* value = 7.72*E* − 8) when compared to normal, while a relatively weak difference is also observed between T-DM group and T-NDM group (mean fold change difference = 0.07, *p* value = 0.15), which implies multiple mechanisms in regulation of* CRYAB*, as well as DNA hypermethylation. The responsive module of* CRYAB* is highly changed in normal and T-NDM (DS = 14.508, *p* value = 3.84*E* − 10). The similar case is* SH3GL2*, deletion of which downregulates tumor growth by modulating* EGFR* signaling [47].

Another interesting case is* S100P*, which has been reported as a key gene in tumor progression in both initial stage and advanced stage in lung adenocarcinoma [60]. The gene shows distinctive expressions among normal, T-DM, and T-NDM. There are nearly no changes existent in gene expression between normal and T-NDM, while in T-DM, upregulation is observed, which implies that the upregulation of* S100P* may be an important step in the early stage of lung adenocarcinomas.

Also some genes are relevant to cancers but lung cancer from literature study (*COL5A2* [51],* SPARCL1* [35],* EFEMP2* [39],* MSR1* [49], and* DOCK2* [61]).* APARCL1* and* DOCK2* have shown downregulation in types of cancer [36, 61], while both of them show downregulated gene expressions in T-DM with high occurrences of DNA hypermethylation. Similar to* CRYAB*,* EFEMP2* shows contrary expression patterns in our observation compared to which in gliomas [39].* EFEMP2* has high occurrences of DNA hypermethylation and downregulated gene expression in totally 20 samples, while 2 samples in T-NDM show little differences when compared to matched normal.* COL5A2* also shows T-DM specific upregulation of gene expression and DNA hypermethylation with high occurrences.

**Figure 4:**
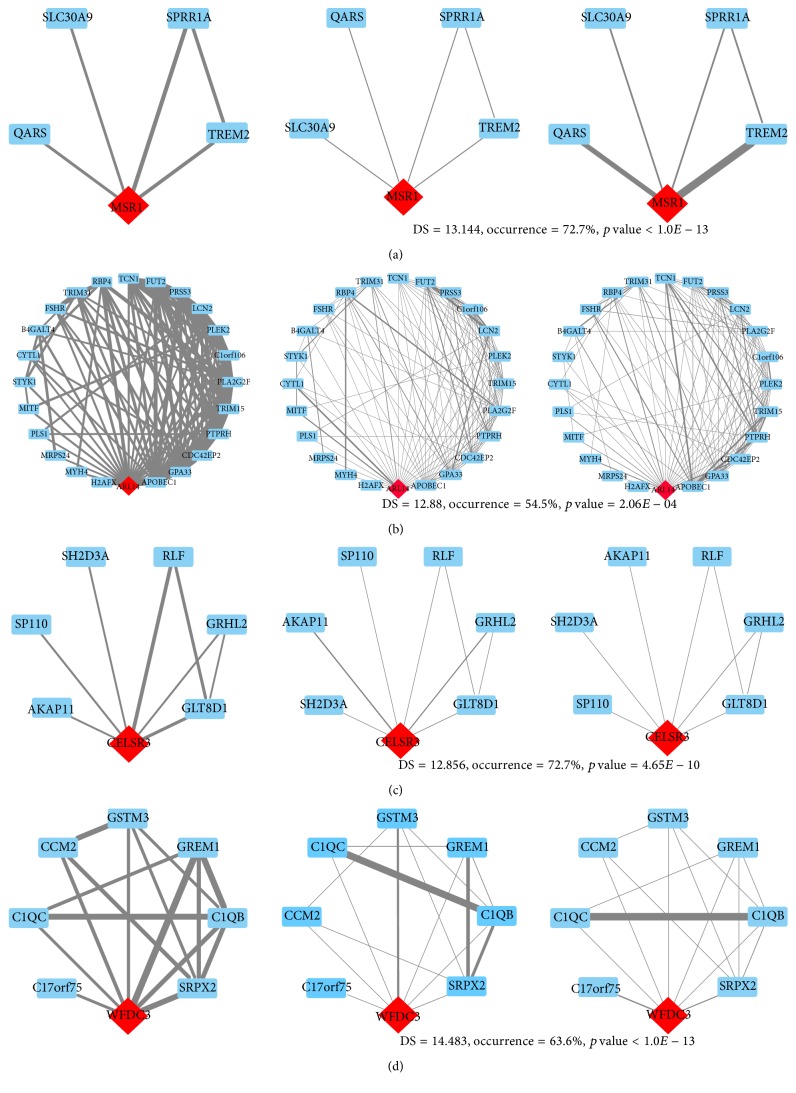
Differential representation of responsive modules for* MSR1*,* ARL14*,* CELSR3*, and* WFDC3* in T-DM (left), normal (middle), and T-NDM (right). Significant changes of responsive modules for identified driver genes (red diamond) imply functional alterations of driver genes in tumorigenesis.

We show the responsive module of* MSR1* in Figure 4(a) as a representation of responsive modules of cancer-related genes. All these genes exhibit significant changes in responsive modules in T-DM when compared to normal and T-NDM.

Besides cancer-related genes, three genes* ARL14*,* CELSR3*, and* WFDC3* are also observed in our list. These three genes show T-DM specific expression changes (Figure 3), and regulatory correlations in responsive modules show significant differences in T-DM when compared to normal and T-NDM (Figures 4(b)–4(d)) which also imply potential roles of the three genes in the tumorigenesis of lung adenocarcinoma.

All top 30 genes show significant changes in responsive modules in T-DM, while detailed information of the top 30 genes and responsive modules are listed in Supplementary File.

## 5. Conclusions

By integration of gene expression and DNA methylation data, we analyzed 22 matched lung adenocarcinoma/nontumor lung pairs for nonsmokers in early stage lung adenocarcinoma. By focusing on differences in gene expression patterns and responsive modules derived from T-DM compared to those in normal and T-NDM, we proposed a pipeline by employing a differential network analysis strategy. Totally, 135 candidate genes are analyzed, and top 30 genes are well studied in this work. All 135 genes are differentially expressed in T-DM when compared to matched normal and T-NDM, while 130 of them show significant changes in regulatory correlations of responsive modules. Literature mining of top 30 genes indicates a high proportion of lung cancer-relevant genes, which implies potential risks of these genes to disturb functions and pathways via differential methylation mechanisms, and further drives the tumorigenesis of lung adenocarcinoma in early stage. In conclusion, we provide a bioinformatics pipeline to identify driver genes with aberrant DNA methylation by fully considering differential expression and network changes in T-DM, normal, and T-NDM. The analysis pipeline can also be employed in identification of driver genes with aberrant DNA methylation of other cancers characterized by paired gene expression and DNA methylation.

## Figures and Tables

**Table 1:** Top 30 genes ranked by differential score in lung adenocarcinoma.

Gene symbol^a^	Differential score	Number of samples in T-DM group^b^	*p* value
**FAM107A [34]**	16.301	20	7.80*E* − 06
SPARCL1 [35, 36]	14.920	20	1.40*E* − 07
TRPC6 [37]	14.649	11	<1.0*E* − 10
**CRYAB [38]**	14.508	12	3.84*E* − 10
WFDC3	14.483	−14	<1.0*E* − 10
EFEMP2 [39]	13.958	20	<1.0*E* − 10
**MX2 [40, 41]**	13.895	−18	2.12*E* − 05
PLA2G4C [42]	13.870	−8	<1.0*E* − 10
ST6GALNAC5 [43]	13.848	9	<1.0*E* − 10
**PLAT [44]**	13.690	8	2.45*E* − 04
**TCF21 [45]**	13.664	22	<1.0*E* − 10
**SOX17 [46]**	13.368	22	<1.0*E* − 10
**SH3GL2 [47]**	13.300	5	<1.0*E* − 10
**MAMDC2 [18]**	13.274	19	4.54*E* − 07
GCNT3 [48]	13.238	−14	<1.0*E* − 10
MSR1 [49]	13.144	−16	<1.0*E* − 10
**PPP1R14D [50]**	13.057	−12	<1.0*E* − 10
COL5A2 [51]	13.045	19	6.67*E* − 04
**PTPRH [52]**	12.967	−16	8.98*E* − 13
HKDC1 [53]	12.961	−20	<1.0*E* − 10
**CDH13 [54]**	12.932	−20	3.34*E* − 04
**CFI [55]**	12.932	5	1.20*E* − 04
ARL14	12.880	−12	2.06*E* − 04
**MMP9 [56]**	12.866	7	<1.0*E* − 10
CELSR3	12.856	16	4.65*E* − 10
**CDO1 [57]**	12.846	22	<1.0*E* − 10
**AGR2 [58]**	12.836	−22	<1.0*E* − 10
**S100P [59, 60]**	12.828	−10	2.29*E* − 04
DOCK2 [61]	12.777	20	2.54*E* − 03
**TNFRSF1B [62]**	12.736	13	<1.0*E* − 10

^a^Bold: gene literature annotated to lung cancer.

^b^−: Gene hypomethylated in samples.

**Table 2:** Functional annotation of driver-responsive network to KEGG signaling pathways (*p* value < 0.01).

Gene symbol	Enriched KEGG signaling pathway	*p* value
SPARCL1	CYTOSOLIC_DNA_SENSING	3.22*E* − 03

TRPC6	PPAR_SIGNALING	9.50*E* − 03
	P53_SIGNALING	9.50*E* − 03
	MTOR_SIGNALING	7.16*E* − 03
	NOTCH_SIGNALING	6.47*E* − 03

EFEMP2	NOTCH_SIGNALING	9.70*E* − 03

MX2	RIG_I_LIKE_RECEPTOR_SIGNALING	9.33*E* − 04

PLA2G4C	PPAR_SIGNALING	9.50*E* − 03
	P53_SIGNALING	9.50*E* − 03
	MTOR_SIGNALING	7.16*E* − 03
	NOTCH_SIGNALING	6.47*E* − 03

ST6GALNAC5	PPAR_SIGNALING	9.50*E* − 03
	P53_SIGNALING	9.50*E* − 03
	MTOR_SIGNALING	7.16*E* − 03
	NOTCH_SIGNALING	6.47*E* − 03

PLAT	TOLL_LIKE_RECEPTOR_SIGNALING	3.09*E* − 03
	NOD_LIKE_RECEPTOR_SIGNALING	1.16*E* − 03
	CYTOSOLIC_DNA_SENSING	9.44*E* − 04
	JAK_STAT_SIGNALING	6.99*E* − 03

TCF21	FC_EPSILON_RI_SIGNALING	4.89*E* − 03

GCNT3	NOTCH_SIGNALING	9.70*E* − 03

MSR1	NOTCH_SIGNALING	9.70*E* − 03

PTPRH	B_CELL_RECEPTOR_SIGNALING	9.80*E* − 03

HKDC1	PPAR_SIGNALING	9.50*E* − 03
	P53_SIGNALING	9.50*E* − 03
	MTOR_SIGNALING	7.16*E* − 03
	NOTCH_SIGNALING	6.47*E* − 03

CDH13	ERBB_SIGNALING	1.55*E* − 03
	T_CELL_RECEPTOR_SIGNALING	2.38*E* − 03

CFI	PPAR_SIGNALING	2.90*E* − 03
	MAPK_SIGNALING	3.47*E* − 03

ARL14	VEGF_SIGNALING	3.93*E* − 03

S100P	HEDGEHOG_SIGNALING	6.47*E* − 04
	TGF_BETA_SIGNALING	1.51*E* − 03

DOCK2	CHEMOKINE_SIGNALING	3.49*E* − 05
	TOLL_LIKE_RECEPTOR_SIGNALING	4.85*E* − 03
	NOD_LIKE_RECEPTOR_SIGNALING	3.27*E* − 05
	T_CELL_RECEPTOR_SIGNALING	5.42*E* − 03
	B_CELL_RECEPTOR_SIGNALING	2.66*E* − 03

TNFRSF1B	NOTCH_SIGNALING	7.06*E* − 03
	FC_EPSILON_RI_SIGNALING	1.24*E* − 03

## References

[1] Zhang H., Cai B. (2003). The impact of tobacco on lung health in China. *Respirology*.

[2] Ferlay J., Autier P., Boniol M., Heanue M., Colombet M., Boyle P. (2007). Estimates of the cancer incidence and mortality in Europe in 2006. *Annals of Oncology*.

[3] Toh C.-K., Gao F., Lim W.-T. (2006). Never-smokers with lung cancer: epidemiologic evidence of a distinct disease entity. *Journal of Clinical Oncology*.

[4] Hu Y., Chen G. (2015). Pathogenic mechanisms of lung adenocarcinoma in smokers and non-smokers determined by gene expression interrogation. *Oncology Letters*.

[5] De Carvalho D., Sharma S., You J. S. (2012). DNA methylation screening identifies driver epigenetic events of cancer cell survival. *Cancer Cell*.

[6] Delpu Y., Cordelier P., Cho W. C., Torrisani J. (2013). DNA methylation and cancer diagnosis. *International Journal of Molecular Sciences*.

[7] De Carvalho D. D., You J. S., Jones P. A. (2010). DNA methylation and cellular reprogramming. *Trends in Cell Biology*.

[8] Meissner A. (2010). Epigenetic modifications in pluripotent and differentiated cells. *Nature Biotechnology*.

[9] Momparler R. L., Bovenzi V. (2000). DNA methylation and cancer. *Journal of Cellular Physiology*.

[10] Kulis M., Esteller M., Zdenko H., Toshikazu U. (2010). DNA methylation and cancer. *Advances in Genetics*.

[11] N. The Cancer Genome Atlas Research (2012). Comprehensive genomic characterization of squamous cell lung cancers. *Nature*.

[12] Balgkouranidou I., Liloglou T., Lianidou E. S. (2013). Lung cancer epigenetics: emerging biomarkers. *Biomarkers in Medicine*.

[13] Kalari S., Pfeifer G. P. (2010). Identification of driver and passenger DNA methylation in cancer by epigenomic analysis. *Advances in Genetics*.

[14] Jjingo D., Conley A. B., Yi S. V., Lunyak V. V., King Jordan I. (2012). On the presence and role of human gene-body DNA methylation. *Oncotarget*.

[15] Yang X., Han H., DeCarvalho D. D., Lay F. D., Jones P. A., Liang G. (2014). Gene body methylation can alter gene expression and is a therapeutic target in cancer. *Cancer Cell*.

[16] Teodoridis J. M., Strathdee G., Brown R. (2004). Epigenetic silencing mediated by CpG island methylation: potential as a therapeutic target and as a biomarker. *Drug Resistance Updates*.

[17] Sigalotti L., Fratta E., Coral S. (2007). Epigenetic drugs as pleiotropic agents in cancer treatment: biomolecular aspects and clinical applications. *Journal of Cellular Physiology*.

[18] Selamat S. A., Chung B. S., Girard L. (2012). Genome-scale analysis of DNA methylation in lung adenocarcinoma and integration with mRNA expression. *Genome Research*.

[19] Tessema M., Yingling C. M., Liu Y. (2014). Genome-wide unmasking of epigenetically silenced genes in lung adenocarcinoma from smokers and never smokers. *Carcinogenesis*.

[20] Karlsson A., Jönsson M., Lauss M. (2014). Genome-wide DNA methylation analysis of lung carcinoma reveals one neuroendocrine and four adenocarcinoma epitypes associated with patient outcome. *Clinical Cancer Research*.

[21] Sato T., Arai E., Kohno T. (2014). Epigenetic clustering of lung adenocarcinomas based on DNA methylation profiles in adjacent lung tissue: its correlation with smoking history and chronic obstructive pulmonary disease. *International Journal of Cancer*.

[22] Zeng X., Zhang X., Zou Q. (2016). Integrative approaches for predicting microRNA function and prioritizing disease-related microRNA using biological interaction networks. *Briefings in Bioinformatics*.

[23] Zou Q., Li J., Song L., Zeng X., Wang G. (2016). Similarity computation strategies in the microRNA-disease network: a survey. *Briefings in Functional Genomics*.

[24] Huber W., Von Heydebreck A., Sültmann H., Poustka A., Vingron M. (2002). Variance stabilization applied to microarray data calibration and to the quantification of differential expression. *Bioinformatics*.

[25] Storey J. D. (2002). A direct approach to false discovery rates. *Journal of the Royal Statistical Society, Series B: Statistical Methodology*.

[26] Lee E., Chuang H.-Y., Kim J.-W., Ideker T., Lee D. (2008). Inferring pathway activity toward precise disease classification. *PLoS Computational Biology*.

[27] Faith J. J., Hayete B., Thaden J. T. (2007). Large-scale mapping and validation of *Escherichia coli* transcriptional regulation from a compendium of expression profiles. *PLoS Biology*.

[28] Daub C. O., Steuer R., Selbig J., Kloska S. (2004). Estimating mutual information using B-spline functions—an improved similarity measure for analysing gene expression data. *BMC Bioinformatics*.

[29] Olsen C., Meyer P. E., Bontempi G. (2009). On the impact of entropy estimation on transcriptional regulatory network inference based on mutual information. *Eurasip Journal on Bioinformatics and Systems Biology*.

[30] Ideker T., Krogan N. J. (2012). Differential network biology. *Molecular Systems Biology*.

[31] Huang D. W., Sherman B. T., Lempicki R. A. (2009). Bioinformatics enrichment tools: paths toward the comprehensive functional analysis of large gene lists. *Nucleic Acids Research*.

[32] Huang D. W., Sherman B. T., Lempicki R. A. (2009). Systematic and integrative analysis of large gene lists using DAVID bioinformatics resources. *Nature Protocols*.

[33] Barabási A.-L., Oltvai Z. N. (2004). Network biology: understanding the cell's functional organization. *Nature Reviews Genetics*.

[34] Pastuszak-Lewandoska D., Czarnecka K. H., Migdalska-Sęk M. (2015). Decreased FAM107A expression in patients with non-small cell lung cancer. *Advances in Experimental Medicine and Biology*.

[35] Xiang Y., Qiu Q., Jiang M. (2013). SPARCL1 suppresses metastasis in prostate cancer. *Molecular Oncology*.

[36] Li P., Qian J., Yu G. (2012). Down-regulated SPARCL1 is associated with clinical significance in human gastric cancer. *Journal of Surgical Oncology*.

[37] Pla A. F., Gkika D. (2013). Emerging role of TRP channels in cell migration: from tumor vascularization to metastasis. *Frontiers in Physiology*.

[38] Qin H., Ni Y., Tong J. (2014). Elevated expression of CRYAB predicts unfavorable prognosis in non-small cell lung cancer. *Medical Oncology*.

[39] Wang L., Chen Q., Chen Z. (2015). EFEMP2 is upregulated in gliomas and promotes glioma cell proliferation and invasion. *International Journal of Clinical and Experimental Pathology*.

[40] Watanabe M., Komeshima N., Nakajima S., Tsuruo T. (1988). MX2, a morpholino anthracycline, as a new antitumor agent against drug-sensitive and multidrug-resistant human and murine tumor cells. *Cancer Research*.

[41] Kobayashi K., Nishioka M., Kohno T. (2004). Identification of genes whose expression is upregulated in lung adenocarcinoma cells in comparison with type II alveolar cells and bronchiolar epithelial cells in vivo. *Oncogene*.

[42] Hartmann C., Johnk L., Sasaki H., Jenkins R. B., Louis D. N. (2002). Novel PLA2G4C polymorphism as a molecular diagnostic assay for 19q loss in human gliomas. *Brain Pathology*.

[43] Bos P. D., Zhang X. H.-F., Nadal C. (2009). Genes that mediate breast cancer metastasis to the brain. *Nature*.

[44] Buccheri G., Ferrigno D. (2002). Lung tumour markers in oncology practice: a study of TPA and CA125. *British Journal of Cancer*.

[45] Smith L. T., Lin M., Brena R. M. (2006). Epigenetic regulation of the tumor suppressor gene TCF21 on 6q23-q24 in lung and head and neck cancer. *Proceedings of the National Academy of Sciences of the United States of America*.

[46] Yin D., Jia Y., Yu Y. (2012). SOX17 methylation inhibits its antagonism of Wnt signaling pathway in lung cancer. *Discovery Medicine*.

[47] Dasgupta S., Jang J. S., Shao C. (2013). SH3GL2 is frequently deleted in non-small cell lung cancer and downregulates tumor growth by modulating EGFR signaling. *Journal of Molecular Medicine (Berlin, Germany)*.

[48] Reticker-Flynn N. E., Bhatia S. N. (2015). Aberrant glycosylation promotes lung cancer metastasis through adhesion to galectins in the metastatic niche. *Cancer Discovery*.

[49] Chen Y., Sullivan C., Peng C. (2011). A tumor suppressor function of the *Msr1* gene in leukemia stem cells of chronic myeloid leukemia. *Blood*.

[50] Lokk K., Vooder T., Kolde R. (2012). Methylation markers of early-stage non-small cell lung cancer. *PLoS ONE*.

[51] Fischer H., Stenling R., Rubio C., Lindblom A. (2001). Colorectal carcinogenesis is associated with stromal expression of COL11A1 and COL5A2. *Carcinogenesis*.

[52] Sato T., Soejima K., Arai E. R. I. (2015). Prognostic implication of PTPRH hypomethylation in non-small cell lung cancer. *Oncology Reports*.

[53] Li G.-H., Huang J.-F. (2014). Inferring therapeutic targets from heterogeneous data: HKDC1 is a novel potential therapeutic target for cancer. *Bioinformatics*.

[54] Toyooka K. O., Toyooka S., Virmani A. K. (2001). Loss of expression and aberrant methylation of the CDH13 (H-cadherin) gene in breast and lung carcinomas. *Cancer Research*.

[55] Okroj M., Hsu Y.-F., Ajona D., Pio R., Blom A. M. (2008). Non-small cell lung cancer cells produce a functional set of complement factor I and its soluble cofactors. *Molecular Immunology*.

[56] Schveigert D., Cicenas S., Bruzas S., Samalavicius N., Gudleviciene Z., Didziapetriene J. (2013). The value of MMP-9 for breast and non-small cell lung cancer patients' survival. *Advances in Medical Sciences*.

[57] Wrangle J., Machida E. O., Danilova L. (2014). Functional identification of cancer-specific methylation of CDO1, HOXA9, and TAC1 for the diagnosis of lung cancer. *Clinical Cancer Research*.

[58] Alavi M., Mah V., Maresh E. L. (2015). High expression of AGR2 in lung cancer is predictive of poor survival. *BMC Cancer*.

[59] Bartling B., Rehbein G., Schmitt W. D., Hofmann H.-S., Silber R.-E., Simm A. (2007). S100A2-S100P expression profile and diagnosis of non-small cell lung carcinoma: impairment by advanced tumour stages and neoadjuvant chemotherapy. *European Journal of Cancer*.

[60] Rehbein G., Simm A., Hofmann H.-S., Silbar R.-E., Bartling B. (2008). Molecular regulation of S100P in human lung adenocarcinomas. *International Journal of Molecular Medicine*.

[61] Nishihara H., Maeda M., Oda A. (2002). DOCK2 associates with CrkL and regulates Rac1 in human leukemia cell lines. *Blood*.

[62] Guan X., Liao Z., Ma H. (2011). TNFRSF1B +676 T>G polymorphism predicts survival of non-Small cell lung cancer patients treated with chemoradiotherapy. *BMC Cancer*.

[63] Kanehisa M., Sato Y., Kawashima M., Furumichi M., Tanabe M. (2016). KEGG as a reference resource for gene and protein annotation. *Nucleic Acids Research*.

[64] Subramanian A., Tamayo P., Mootha V. K. (2005). Gene set enrichment analysis: a knowledge-based approach for interpreting genome-wide expression profiles. *Proceedings of the National Academy of Sciences of the United States of America*.

